# An L1CAM-Positive Fibroblast-Associated Stromal State Is Associated with Reduced T/NK Cytotoxicity Features in Colorectal Cancer

**DOI:** 10.3390/biomedicines14091900

**Published:** 2026-08-26

**Authors:** Jian Zou, Yingna Cai, Miao Sun, Lingyu Zhang, Chengkuan Zhao, Xiaolong Wu, Yi Liu, Jinyan Chen, Yuming Liang, Xiaoxu Zhao, Shuyao Zhang

**Affiliations:** 1 Department of Pharmacy, Guangzhou Red Cross Hospital of Jinan University, Guangzhou 510220, China; 2 State Key Laboratory of Bioactive Molecules and Druggability Assessment, International Cooperative Laboratory of Traditional Chinese Medicine Modernization and Innovative Drug Discovery of Chinese Ministry of Education, Guangzhou City Key Laboratory of Precision Chemical Drug Development, School of Pharmacy, Jinan University, Guangzhou 510632, China; 3 Department of Pharmacology, Shantou University Medical College, Shantou 515041, China; 4 College of Pharmacy, Jinan University, Guangzhou 510632, China; 5 General Surgery, The Fifth Affiliated Hospital of Jinan University, Heyuan 510632, China

**Keywords:** *L1CAM*, colorectal cancer, fibroblast, tumor microenvironment, T/NK cells

## Abstract

**Background/Objectives:** L1 cell adhesion molecule (*L1CAM*) has been implicated in colorectal cancer progression, but its cellular source and immune microenvironmental context remain incompletely defined. This study aimed to characterize the expression pattern, cellular localization and immune-associated features of *L1CAM* in colorectal cancer using public bulk and single-cell datasets. **Methods:** TCGA, GTEx and CPTAC/UALCAN datasets were used to assess *L1CAM* expression, protein abundance and clinical associations across cancers, with a focus on colorectal cancer. Single-cell RNA sequencing data from GSE178341 were analyzed to identify *L1CAM*-expressing cell populations, characterize transcriptional programs and evaluate sample-level associations with immune-cell composition and T/NK cytotoxicity signatures. Candidate ligand-receptor interactions and conceptual mod el-based sensitivity analyses were used to explore stromal-immune communication axes. Supplementary external single-cell datasets were analyzed exploratorily. **Results:** In colorectal cancer, *L1CAM* showed tumor-normal expression differences and was associated with progression-free interval, but not overall survival. Single-cell analysis detected *L1CAM* mainly in rare fibroblast and epithelial subsets rather than T/NK cells. L1CAM-positive fibroblasts showed neural-like, wound-response and matrix-remodeling transcriptional features. In the main cohort, L1CAM-Fib+ samples showed lower T/NK cytotoxicity transcriptional signatures, whereas supplementary external datasets did not reproduce the same direction of association. Candidate communication analyses nominated Galectin, HLA-E, TGFB and extracellular matrix-related axes. **Conclusions:** These findings suggest that L1CAM-positive fibroblast-associated stromal states provide a dataset-specific exploratory context for interpreting L1CAM-associated immune features in colorectal cancer. The results should be considered hypothesis-generating and require spatial and functional validation.

## 1. Introduction

Colorectal cancer (CRC) remains a major cause of cancer-related morbidity and mortality worldwide [1,2]. Although surgery, chemotherapy, targeted therapy and immunotherapy have improved clinical management, recurrence and metastasis remain common causes of treatment failure [3,4]. Immune checkpoint blockade has produced durable responses in mismatch repair-deficient or microsatellite instability-high CRC, but most patients with proficient mismatch repair or microsatellite-stable tumors derive limited benefit [5]. These clinical observations highlight the need to better understand the tumor microenvironment, including stromal and immune-cell states that may shape tumor progression and immune responsiveness.

The tumor microenvironment of CRC contains diverse epithelial [6,7], stromal [8,9], endothelial and immune-cell populations [10,11]. Among these components, cancer-associated fibroblasts have been increasingly recognized as heterogeneous stromal cells that can participate in extracellular matrix remodeling [12,13], wound-response programs [12,14], immune-cell recruitment [15,16] and immune modulation [17]. However, fibroblast states are not uniform, and the biological meaning of a marker can depend strongly on the cell type in which it is expressed [7,18,19]. Therefore, resolving the cellular source of tumor-associated genes is essential for interpreting bulk transcriptomic signals and their relationship with immune features [7].

L1 cell adhesion molecule (*L1CAM*) is a neural cell adhesion molecule [20,21] that has been implicated in several cancer-related processes [21,22], including invasion [23,24], migration [25,26], epithelial plasticity, metastasis [27] and poor prognosis [28,29]. In CRC, previous studies have associated *L1CAM* expression with aggressive tumor behavior and unfavorable clinical outcomes [30,31,32]. These findings have largely emphasized tumor-cell-intrinsic roles of *L1CAM*. However, bulk tumor measurements cannot distinguish whether L1CAM-associated signals arise from malignant epithelial cells, stromal cells or other components of the tumor microenvironment. The immune context of *L1CAM* expression in CRC also remains incompletely defined.

In this study, we integrated pan-cancer transcriptomic data, protein-level data, clinical follow-up data and public CRC single-cell RNA sequencing datasets to characterize the expression pattern, cellular localization and immune microenvironmental context of L1CAM. The aim was not to establish L1CAM as a validated therapeutic target, but to clarify the cellular and sample-level context in which L1CAM signals appear in CRC and to nominate candidate stromal-immune associations for future validation.

## 2. Materials and Methods

### 2.1. Data Sources

This study used publicly available de-identified data and did not collect new human biopsies. TCGA expression, phenotype and clinical follow-up data were obtained from public cancer genomics resources and UCSC Xena. For cancer types without TCGA normal samples, GTEx normal tissues were supplemented using the UCSC Xena Toil recomputed TCGA/GTEx TPM matrix. Protein-level comparisons used *L1CAM* total-protein data provided by the CPTAC module in UALCAN. The main single-cell RNA sequencing analysis used the GEO dataset GSE178341; supplementary external exploratory analyses used the GEO datasets GSE231559 and GSE188711.

### 2.2. Pan-Cancer mRNA Expression Analysis

*L1CAM* mRNA expression was compared between tumor and normal samples within each cancer type. TCGA samples were classified as primary tumor or normal tissue according to sample type codes, and cancer names were unified as TCGA abbreviations. Cancer types with TCGA normal samples were used for tumor-normal comparison; cancer types with insufficient TCGA normal samples were supplemented with GTEx normal tissues. Differences between two groups were assessed using the Wilcoxon rank-sum test.

### 2.3. Protein, AJCC Stage and Survival Analyses

*L1CAM* protein levels were compared between tumor and normal tissues using UALCAN/CPTAC summary data. AJCC stage analysis included only cancer types with ajcc_pathologic_tumor_stage annotations, at least 20 valid staged samples and at least two AJCC I-IV stage groups. Stage associations were evaluated using Spearman correlation and the Kruskal-Wallis test, with multiple testing corrected by the Benjamini–Hochberg method. For survival analysis, samples within each cancer type were divided into high- and low-expression groups by the median L1CAM expression level, and OS and PFI were evaluated separately. Cox proportional hazards models were used to estimate hazard ratios (HRs) and *p* values, and the log-rank test was used for Kaplan–Meier curve comparisons.

### 2.4. GSE178341 Single-Cell Data Processing

The 10x h5 matrices, cluster annotation table and meta-table metadata from GSE178341 were used for the main single-cell analysis. Cell types were based on the top-level annotations provided by the original dataset and were uniformly mapped to categories including Epithelial, T/NK, B, Plasma, Myeloid, Fibroblast, Mast and Other. L1CAM-positive cells were defined using a detection-based criterion as cells with *L1CAM* expression greater than 0 after log1p normalization. This detection-based definition was used because *L1CAM* expression was sparse in the single-cell–matrix. UMAP coordinates and cell-type annotations were used to display the distribution of *L1CAM* across the cellular atlas.

### 2.5. Differential Expression and GO Enrichment Analysis of L1CAM-Positive Cells

Differential expression analysis between *L1CAM*-positive and L1CAM-negative cells was performed separately in fibroblasts and epithelial cells. In each cell class, up to 1500 L1CAM-negative cells were randomly sampled as controls to reduce imbalance caused by the much larger number of L1CAM-negative cells while retaining sufficient control cells for differential expression testing; the random seed was set to 397. Genes were retained if the fraction of expressing cells was at least 0.01 in either group, or if the mean log1p-normalized expression was at least 0.005 in either group. Differential expression was assessed using the Wilcoxon rank-sum test, and *p* values were corrected by the Benjamini–Hochberg method. Significantly upregulated genes were defined as FDR < 0.05 and log2FC > 0.25, and significantly downregulated genes were defined as FDR < 0.05 and log2FC < −0.25. GO biological process enrichment of upregulated genes was performed using clusterProfiler and org.Hs.eg.db, with the genes entering differential expression analysis used as the background and *p* values corrected by the Benjamini–Hochberg method.

### 2.6. Sample-Level L1CAM-Positive Fibroblast and Immune Signature Analysis

Sample-level analyses included only tumor-derived samples. For each tumor sample, we calculated the proportions of major immune cell types, fibroblast counts, L1CAM-positive fibroblast counts and the proportion of L1CAM-positive fibroblasts. Because L1CAM-positive fibroblasts represented a rare-cell population, tumor samples were grouped using a presence/absence detection rule. Samples with fewer than 20 recovered fibroblasts were excluded from L1CAM-Fib group comparisons. A sample was defined as L1CAM-Fib+ if at least one L1CAM-positive fibroblast was detected; otherwise, it was defined as L1CAM-Fib-. The T/NK cytotoxicity signature was calculated as the mean log1p-normalized expression of GZMB, *PRF1*, *NKG7*, *GNLY*, GZMA and GZMK. T/NK exhaustion, myeloid inflammation and myeloid immunosuppression signatures were calculated using predefined gene sets. Associations between the proportion of L1CAM-positive fibroblasts and immune-cell proportions or signature scores were assessed using Spearman correlation. Comparisons between L1CAM-Fib+ and L1CAM-Fib- groups were performed using the Wilcoxon rank-sum test, with multiple testing corrected by the Benjamini–Hochberg method.

### 2.7. L1CAM-Fib+ Grouping Sensitivity Analysis

Two alternative grouping strategies were used to evaluate the sensitivity of the L1CAM-Fib+ definition. In the stricter count-based analysis, samples with at least two L1CAM-positive fibroblasts were classified as L1CAM-Fib+, samples with no L1CAM-positive fibroblasts were classified as L1CAM-Fib-, and samples with one L1CAM-positive fibroblast were treated as borderline and excluded from this comparison. In the proportion-based analysis, samples were grouped using the median L1CAM-positive fibroblast proportion among samples with detectable L1CAM-positive fibroblasts. Group differences in T/NK cytotoxicity scores were tested using two-sided Wilcoxon rank-sum tests. The continuous association between L1CAM-positive fibroblast proportion and T/NK cytotoxicity score was assessed using Spearman correlation analysis.

### 2.8. Candidate Ligand–Receptor Communication Analysis

Candidate communication analysis focused on predefined fibroblast-to-T/NK ligand–receptor pairs covering Galectin, MHC/HLA-E, TGFB, chemokine, cytokine, checkpoint, ECM-integrin, Semaphorin and Angiogenic categories. For each ligand–receptor pair, the sample-level interaction score was calculated as the product of mean ligand expression in source cells and mean receptor expression in target cells. The relationship between L1CAM-positive fibroblast-derived ligands and sample-level T/NK cytotoxicity signature was assessed using Spearman correlation.

### 2.9. Signature-Based In Silico Attenuation Sensitivity Analysis

In L1CAM-Fib+ samples, selected candidate ligand values derived from L1CAM-positive fibroblasts were attenuated in silico. The attenuated candidate axes included LGALS1-PTPRC, LGALS3-PTPRC, *HLA-E*-KLRC1, TGFB1-TGFBR2, FN1-ITGB1 and COL1A1-ITGA1. In the primary analysis, candidate ligand expression was multiplied by 0.5, corresponding to 50% attenuation. Additional sensitivity analyses used multipliers of 0.75, 0.5 and 0.25, corresponding to 25%, 50% and 75% attenuation. Candidate ligand–receptor scores were recalculated at each attenuation level. The integrated ligand–receptor score was calculated as the mean of candidate axis scores standardized using the baseline mean and standard deviation. Predicted T/NK cytotoxicity values were estimated using the linear model T_NK_cytotoxicity ~ suppressive_LR_score_baseline. Baseline and attenuated scores were compared using two-sided paired Wilcoxon signed-rank tests.

### 2.10. Supplementary External Exploratory Analysis of GSE231559 and GSE188711

For consistency across the supplementary external datasets, cell identities in GSE231559 and GSE188711 were inferred using broad marker scores. Marker sets were defined as *EPCAM*/*KRT8*/*KRT18*/*KRT19* for epithelial cells, *COL1A1*/*COL1A2*/*COL3A1*/*DCN*/*LUM*/*PDGFRA* for fibroblasts, and *CD3D*/*CD3E*/*CD8A*/*NKG7*/*GNLY*/*PRF1* for T/NK cells. Cells were assigned to the cell type with the highest marker score when the maximum score exceeded 0.10; cells below this threshold were assigned to Other. Tumor samples with at least 10 fibroblasts and 10 T/NK cells were retained for correlation analysis. Associations between the proportion of L1CAM-positive fibroblasts and T/NK cytotoxicity scores were assessed using Spearman correlation.

### 2.11. Statistical Analysis and Software

Unless otherwise specified, all statistical tests were two-sided. Continuous variables between two groups were compared using the Wilcoxon rank-sum test or paired Wilcoxon test, as appropriate. Correlations were assessed using Spearman correlation analysis. Survival analyses were performed using Cox proportional hazards models and log-rank tests. Multiple testing was corrected using the Benjamini–Hochberg method. Analyses were performed using R 4.5.1 and Python 3.12.13. Major R packages included ggplot2 4.0.1, dplyr 1.1.4, readr 2.1.6, tidyr 1.3.1, survival 3.8.3, survminer 0.5.1, data.table 1.17.8, cowplot 1.2.0, clusterProfiler 4.16.0 and org.Hs.eg.db 3.21.0. Major Python packages included numpy 2.3.5, pandas 2.2.3, scipy 1.17.1, h5py 3.16.0 and scikit-learn 1.9.0.

## 3. Results

### 3.1. L1CAM Is Reduced in COAD Tumors but High Intra-Tumoral Expression Associates with Shorter PFI

**Figure 1:**
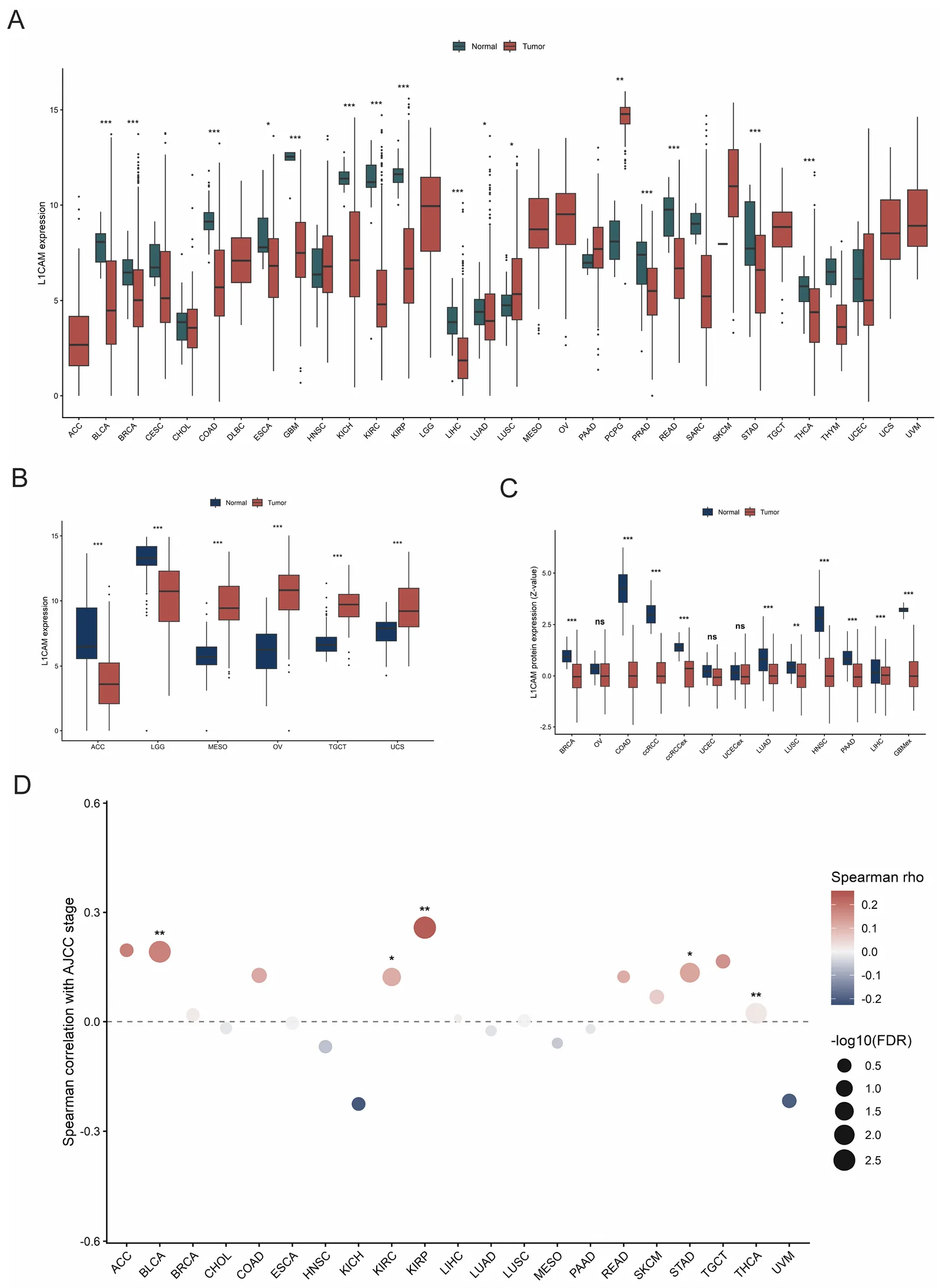
**Pan-cancer expression and clinical-stage associations of *L1CAM*.** (**A**) *L1CAM* mRNA expression in tumor and normal tissues across TCGA cancer types with available normal samples. Cancer types are shown using TCGA abbreviations. (**B**) *L1CAM* expression comparison in cancer types with insufficient TCGA normal samples after supplementation with GTEx normal tissues. (**C**) *L1CAM* total protein levels in tumor and normal tissues based on UALCAN/CPTAC data. (**D**) Association between *L1CAM* expression and AJCC pathological stage in cancer types with available stage annotations. Box plots show medians and interquartile ranges. Two-group comparisons were performed using two-sided Wilcoxon rank-sum tests. Stage associations were assessed using Spearman correlation analysis, and multiple testing across cancer types was corrected using the Benjamini–Hochberg method. Asterisks indicate statistical significance: * *p* < 0.05, ** *p* < 0.01 and *** *p* < 0.001.

**Figure 2:**
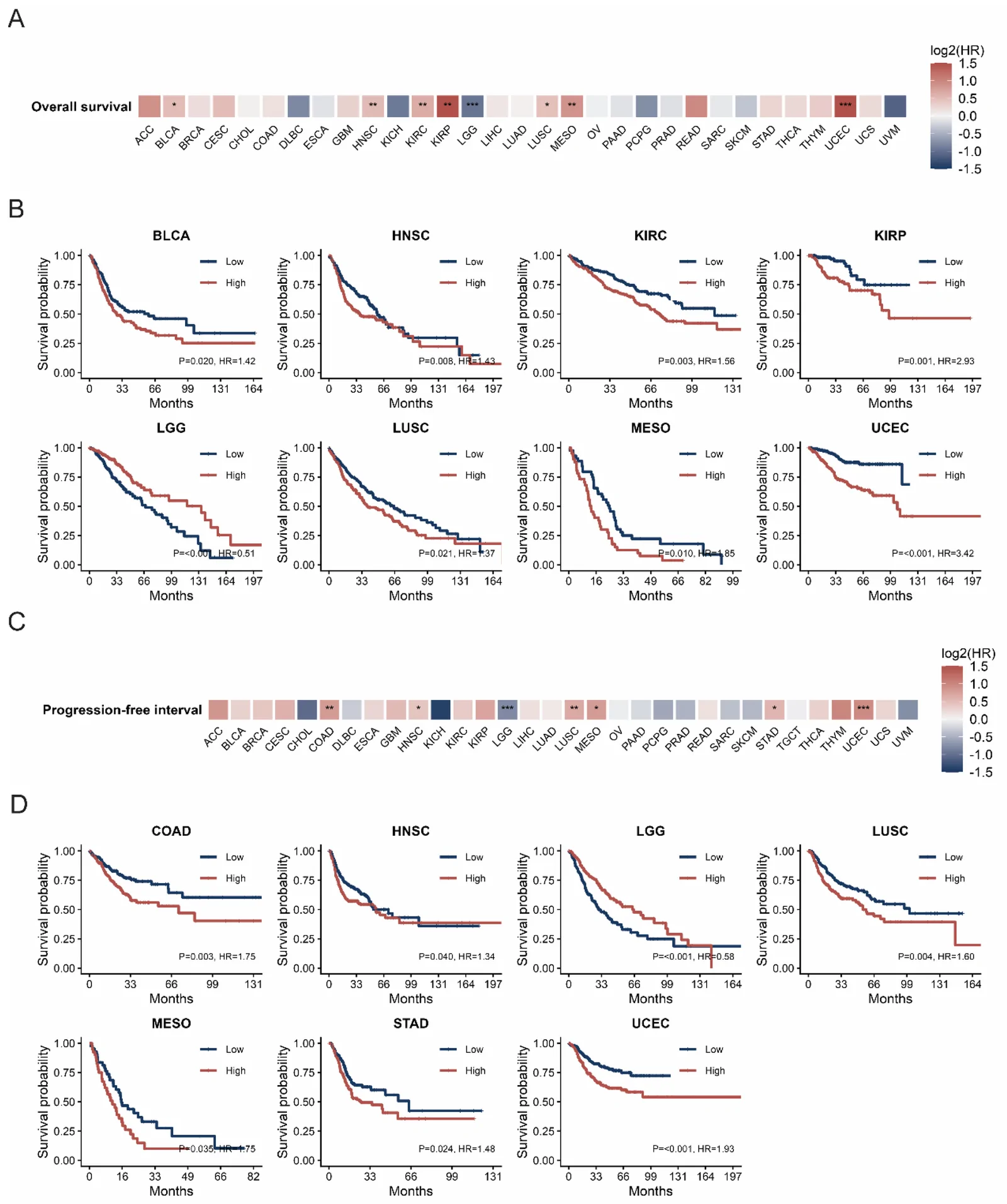
***L1CAM* survival associations vary by cancer type and clinical endpoint.** (**A**) Overall survival (OS) heat map based on TCGA clinical follow-up data. (**B**) OS Kaplan–Meier curves for selected cancer types. (**C**) Progression-free interval (PFI) heat map based on TCGA clinical follow-up data. (**D**) PFI Kaplan–Meier curves for selected cancer types. Samples were grouped according to *L1CAM* expression levels. Hazard ratios were estimated using Cox proportional hazards models, survival curves were compared using the log-rank test, and multiple testing across cancer types was corrected using the Benjamini–Hochberg method. Asterisks indicate statistical significance: * *p* < 0.05, ** *p* < 0.01 and *** *p* < 0.001.

*L1CAM* expression was lower in COAD tumors than in normal tissues, whereas higher intra-tumoral expression was associated with increased PFI risk. In the TCGA cohort, *L1CAM* mRNA expression was lower in 452 tumors than 41 normal samples, with median values of 5.695 and 9.130, respectively (*p* = 1.65 × 10^−16^; Figure 1A). For cancer types with insufficient TCGA normal tissues, GTEx-supplemented comparisons were used to evaluate tumor-normal differences (Figure 1B). CPTAC/UALCAN protein data showed the same direction, with lower *L1CAM* protein levels in 97 tumors than 100 normal samples (median 0 versus 4.245; *p* = 2.49 × 10^−71^; Figure 1C). Pan-cancer survival maps summarized the association between *L1CAM* expression and OS or PFI across tumor types (Figure 2A,C). Survival analysis showed that high *L1CAM* expression was associated with shorter PFI (HR = 1.750, Cox *p* = 0.003; Figure 2D), but not with OS (HR = 1.120, Cox *p* = 0.579; Figure 2B). AJCC stage analysis showed only a weak association between *L1CAM* expression and stage, which did not remain significant after correction across cancer types (rho = 0.127, *p* = 0.008; Figure 1D). Together, these results suggest that tumor-normal differences and within-tumor progression-associated signals reflect distinct aspects of *L1CAM* biology in COAD.

### 3.2. L1CAM Is Detected in Rare Fibroblast and Epithelial Subsets

**Figure 3:**
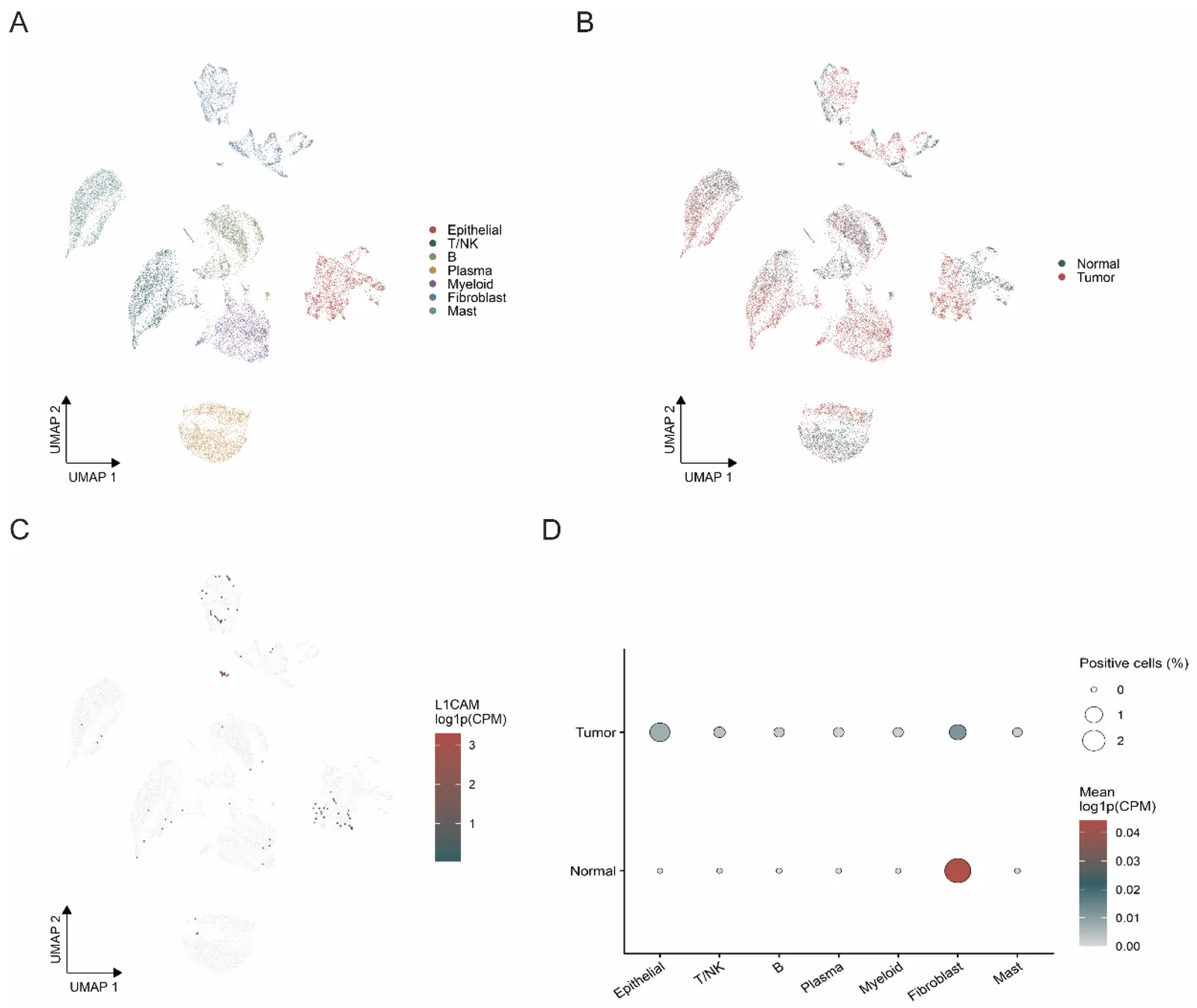
***L1CAM* is detected in rare fibroblast and epithelial subsets in colorectal cancer single-cell data.** (**A**) UMAP visualization of major cell types in the GSE178341 single-cell RNA-seq dataset. (**B**) Distribution of cells from tumor and normal tissues. (**C**) *L1CAM* expression projected onto the UMAP embedding. (**D**) Proportion and mean expression level of L1CAM-positive cells across cell types and tissue sources.

Single-cell analysis detected *L1CAM* in rare fibroblast and epithelial subsets, with lower detection in T/NK cells. Among 24,000 cells in GSE178341, only 113 cells expressed *L1CAM*, accounting for 0.471% of all cells. After stratification by cell type and tissue source, L1CAM-positive cells accounted for 2.960% of normal fibroblasts (38/1284), 0.888% of tumor fibroblasts (19/2139), 1.511% of tumor epithelial cells (35/2317) and 0.258% of tumor T/NK cells (7/2710; Figure 3A–D). These data identify fibroblasts and epithelial cells, rather than T/NK cells, as the main cellular contexts of *L1CAM* expression in the colorectal cancer single-cell atlas.

### 3.3. L1CAM-Positive Fibroblasts Show Neural-like and Wound-Response Programs

**Figure 4:**
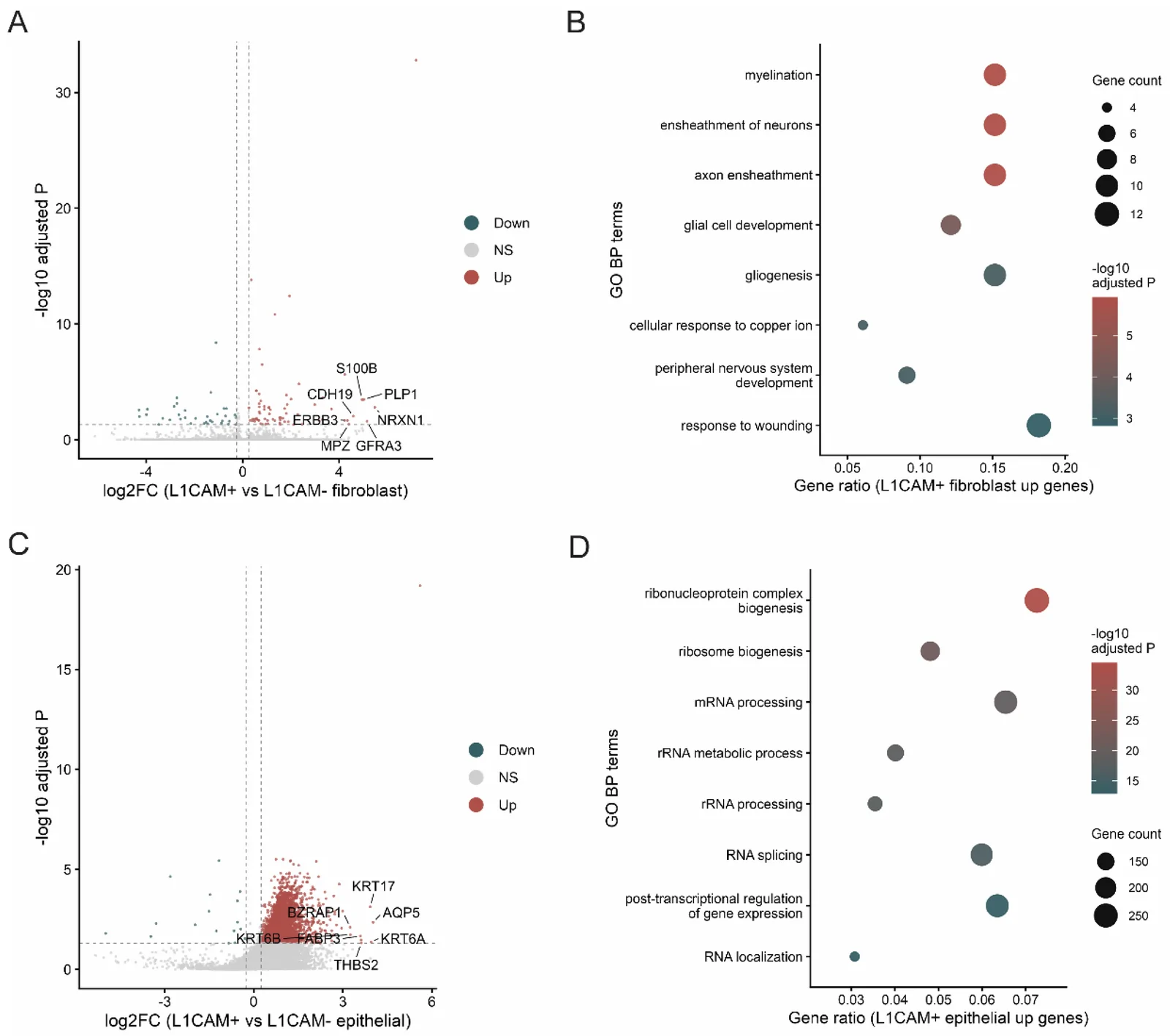
**L1CAM-positive fibroblasts and epithelial cells show distinct transcriptional programs.** (**A**) Differentially expressed genes between L1CAM-positive and L1CAM-negative fibroblasts. (**B**) Gene Ontology (GO) biological process enrichment of genes upregulated in L1CAM-positive fibroblasts. (**C**) Differentially expressed genes between L1CAM-positive and L1CAM-negative epithelial cells. (**D**) GO biological process enrichment of genes upregulated in L1CAM-positive epithelial cells. Differential expression analyses were performed within each broad cell class. Enrichment analyses were based on upregulated gene sets, and multiple testing was corrected using the Benjamini–Hochberg method. Dashed lines in the volcano plots indicate log2 fold-change thresholds of ±0.25 and a nominal *p* value threshold of 0.05.

L1CAM-positive fibroblasts showed neural-like and wound-response-related transcriptional features. Comparison of 57 L1CAM-positive fibroblasts with 1500 L1CAM-negative control fibroblasts identified 69 upregulated genes and 35 downregulated genes (Figure 4A). Upregulated genes included *L1CAM*, *VIM*, *CLU*, *CD9*, *CALD1*, *S100A10*, *RHOB*, *GPM6B*, *ALDH1A1* and *ANXA2*. GO enrichment of upregulated genes was concentrated in myelination, ensheathment of neurons, axon ensheathment, glial cell development, gliogenesis, peripheral nervous system development and response to wounding (Figure 4B). By contrast, L1CAM-positive epithelial cells showed enrichment for ribonucleoprotein complex biogenesis, ribosome biogenesis, mRNA processing and RNA splicing (Figure 4C,D). Thus, L1CAM-positive fibroblasts and epithelial cells represent transcriptionally distinct L1CAM-positive states in the colorectal cancer single-cell atlas.

### 3.4. L1CAM-Fib+ Samples Show Reduced T/NK Cytotoxicity Signatures

**Figure 5:**
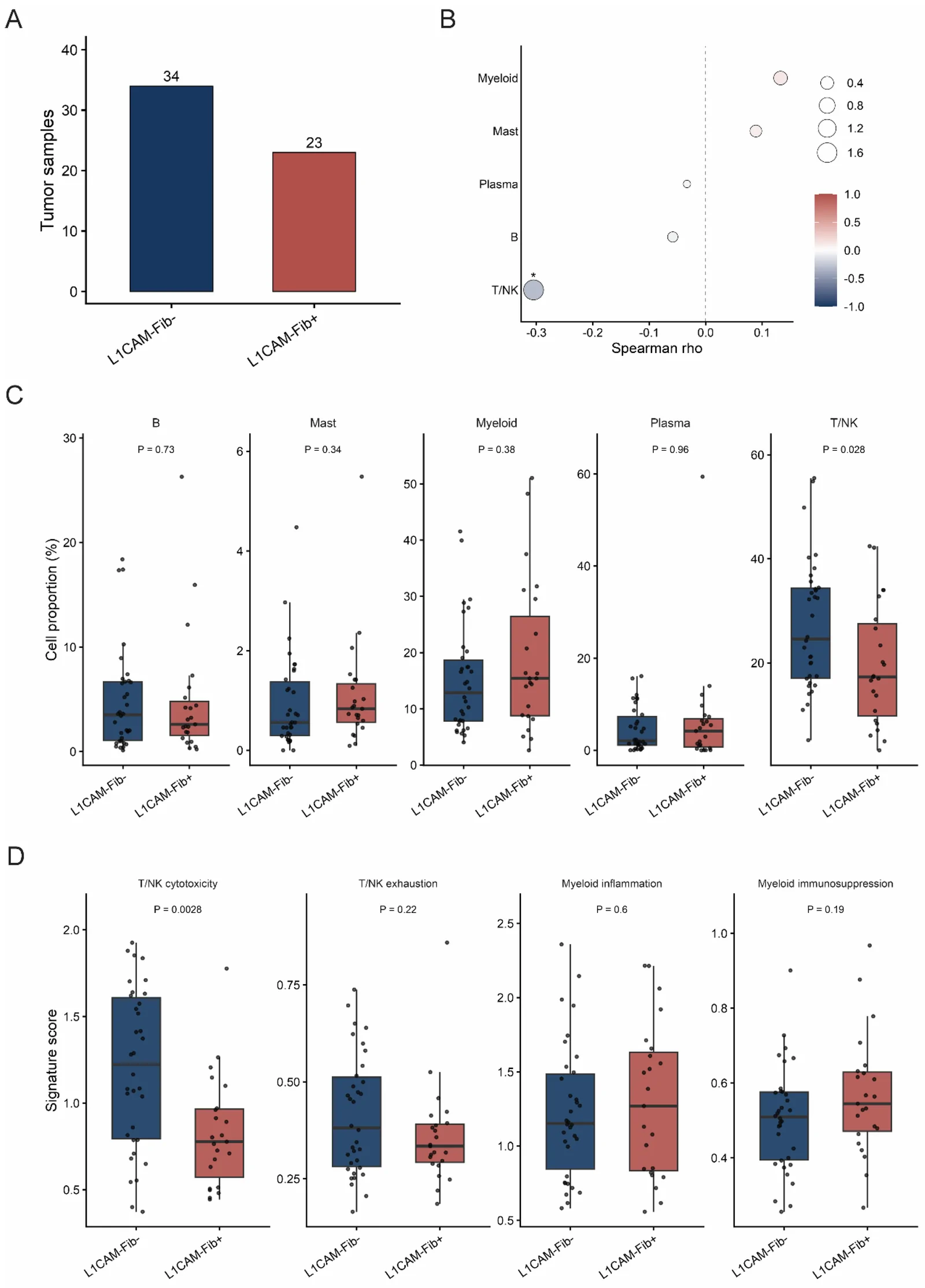
**L1CAM-Fib+ tumor samples show lower T/NK cytotoxicity transcriptional features.** (**A**) Numbers of L1CAM-Fib+, L1CAM-Fib- and low-fibroblast-recovery tumor samples in GSE178341. (**B**) Sample-level correlations between the proportion of L1CAM-positive fibroblasts and immune-cell composition. (**C**) Immune-cell proportions in L1CAM-Fib+ and L1CAM-Fib- samples. (**D**) Immune-related signature scores in L1CAM-Fib+ and L1CAM-Fib- samples. Correlations were assessed using Spearman tests. Group comparisons were performed using two-sided Wilcoxon rank-sum tests, and multiple testing was corrected using the Benjamini–Hochberg method. * *p* < 0.05.

Reduced T/NK cytotoxicity was the most consistent immune feature of L1CAM-Fib+ samples. In GSE178341, 64 tumor-derived samples were included in sample-level immune microenvironment analysis. After filtering for fibroblast recovery, 57 samples were assigned to groups, including 34 L1CAM-Fib- samples and 23 L1CAM-Fib+ samples; 7 samples were excluded from group comparisons because of insufficient fibroblast recovery (Figure 5A). The proportion of L1CAM-positive fibroblasts was negatively correlated with the proportion of T/NK cells, although this association was attenuated after multiple-testing correction (rho = −0.305, *p* = 0.021, FDR = 0.105; Figure 5B). Group comparison showed a consistent direction, with lower T/NK-cell proportions in L1CAM-Fib+ than in L1CAM-Fib- samples (0.195 versus 0.272; *p* = 0.028, FDR = 0.141; Figure 5C). The reduction in T/NK cytotoxicity score was more consistent: mean scores were 0.827 in L1CAM-Fib+ samples and 1.202 in L1CAM-Fib- samples (Wilcoxon *p* = 0.003, FDR = 0.011), and correlation analysis supported a negative association (rho = −0.377, *p* = 0.004, FDR = 0.015; Figure 5D). These data indicate a dataset-specific exploratory association between L1CAM-Fib+ tumors and lower T/NK cytotoxicity transcriptional features in GSE178341. Grouping sensitivity analyses supported the same overall direction but showed threshold-dependent statistical strength. When L1CAM-Fib+ was defined using a stricter threshold of at least two L1CAM-positive fibroblasts, T/NK cytotoxicity remained lower in the L1CAM-Fib+ group, although the association was weaker (*p* = 0.074; Supplementary Figure S2D,E). A proportion-based cutoff retained the same direction (*p* = 0.039), and continuous analysis showed a negative correlation between L1CAM-positive fibroblast proportion and T/NK cytotoxicity score (rho = −0.377, *p* = 0.0038; Supplementary Figure S2F).

### 3.5. Ligand–Receptor Analysis Nominates Immune-Regulatory and Matrix-Remodeling FIBROBLAST-to-T/NK Axes

**Figure 6:**
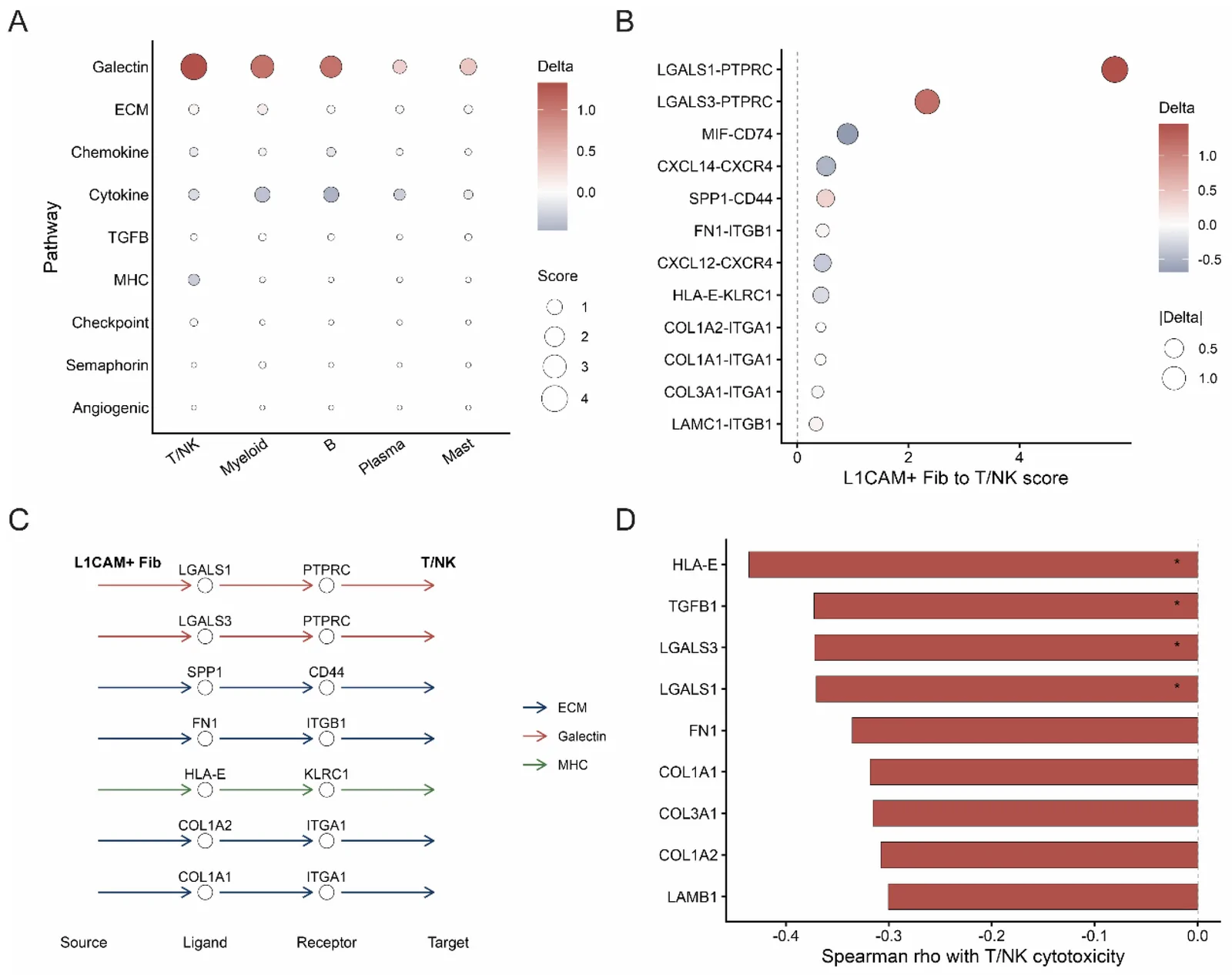
**L1CAM-positive fibroblasts nominate candidate stromal-immune communication axes.** (**A**) Ligand–receptor pathway categories involving L1CAM-positive fibroblasts and immune-cell populations. Dot size denotes interaction score and color denotes relative change. (**B**) Representative candidate ligand–receptor pairs from L1CAM-positive fibroblasts to T/NK cells. Dot size denotes absolute change and color denotes relative change. (**C**) Network view of selected L1CAM-positive fibroblast-to-T/NK ligand–receptor pairs, grouped by pathway category. (**D**) Spearman correlations between fibroblast- or L1CAM-positive fibroblast-derived ligands and sample-level T/NK cytotoxicity scores. Asterisks denote statistically significant correlations.

Ligand–receptor analysis nominated candidate fibroblast-to-T/NK communication axes related to immune regulation and matrix remodeling. Candidate pathways included Galectin, MHC/HLA-E, TGFB, chemokine, cytokine, checkpoint and ECM-related categories (Figure 6A). High-scoring interactions included LGALS1-PTPRC, LGALS3-PTPRC, MIF-CD74, CXCL14-CXCR4, SPP1-CD44, FN1-ITGB1, *HLA-E*-KLRC1 and *COL1A1*/2-ITGA1 (Figure 6B,C). Several L1CAM-positive fibroblast-derived ligands were negatively correlated with sample-level T/NK cytotoxicity scores (Figure 6D). Supplementary marker-based analyses of GSE231559 and GSE188711 showed detectable L1CAM-positive fibroblast-associated signals, but their relationship with T/NK cytotoxicity was not directionally consistent with the main cohort (Supplementary Figure S1A–F). In GSE231559, the proportion of L1CAM-positive fibroblasts was positively correlated with T/NK cytotoxicity score (rho = 0.616, *p* = 0.033; Supplementary Figure S1A–C), whereas GSE188711 showed a weaker and non-significant positive trend (Supplementary Figure S1D–F). Thus, the ligand–receptor results prioritize candidate stromal-immune axes, while the external dataset highlights context dependence of the L1CAM-positive fibroblast and T/NK cytotoxicity relationship.

### 3.6. In Silico Attenuation Supports Directional Consistency of Candidate Axes

**Figure 7:**
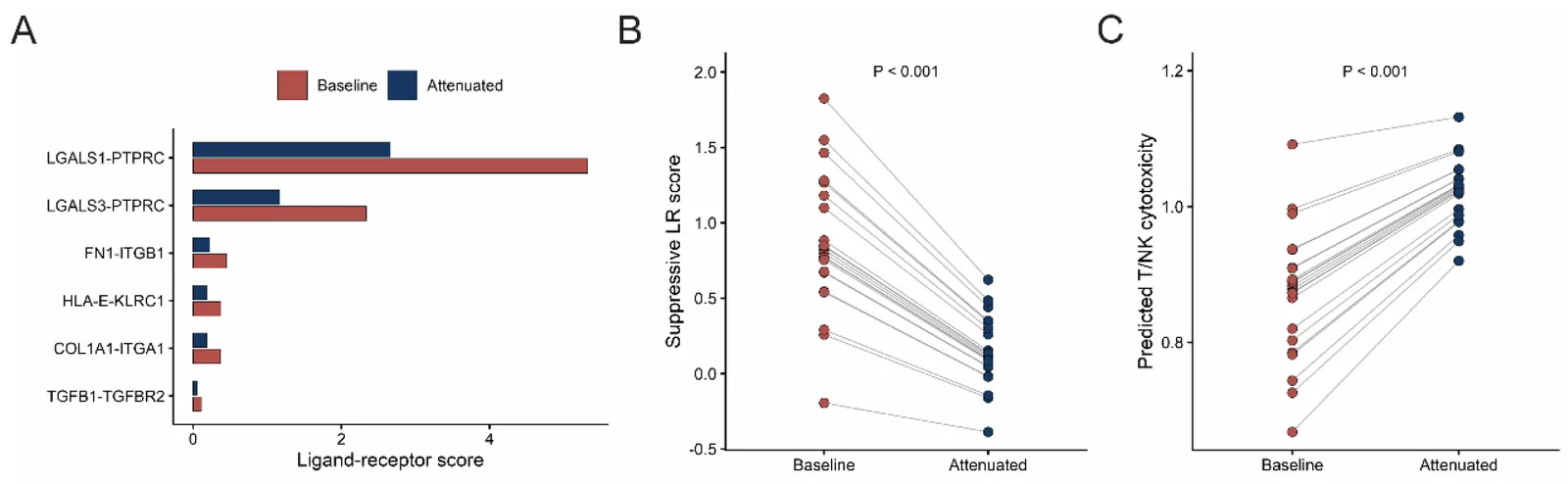
**Conceptual model-based attenuation analysis of candidate stromal-immune axes.** (**A**) Candidate ligand–receptor axis scores before and after 50% attenuation. (**B**) Integrated candidate ligand–receptor score before and after 50% attenuation. (**C**) Model-estimated T/NK cytotoxicity score before and after 50% attenuation. In L1CAM-Fib+ samples, selected L1CAM-positive fibroblast-derived ligand values were set to 50% of the original value within a conceptual model-based sensitivity analysis. Paired comparisons were performed using two-sided Wilcoxon signed-rank tests. This analysis does not represent experimental perturbation, ligand blockade or causal evidence.

A conceptual model-based sensitivity analysis was used to assess whether the candidate fibroblast-to-T/NK axes were directionally consistent with the T/NK cytotoxicity pattern observed in GSE178341. At 50% attenuation, candidate ligand–receptor axis scores decreased after reducing selected L1CAM-positive fibroblast-derived ligand values (Figure 7A). The integrated candidate ligand–receptor score decreased from 0.857 to 0.139 (Figure 7B), whereas model-estimated T/NK cytotoxicity increased from 0.871 to 1.022 (Figure 7C). Sensitivity analyses using 25%, 50% and 75% attenuation showed a monotonic decrease in the integrated candidate ligand–receptor score and a corresponding increase in model-estimated T/NK cytotoxicity (Supplementary Figure S2A–C). These analyses support directional consistency within a conceptual computational model but do not represent experimental perturbation, ligand blockade or causal evidence.

## 4. Discussion

In this study, we observed that *L1CAM* in colorectal cancer was mainly detected as a rare signal in fibroblast and epithelial subsets, and that tumors containing L1CAM-positive fibroblasts showed lower T/NK cytotoxicity transcriptional features in the main single-cell cohort. Pan-cancer and protein-level analyses suggested *L1CAM* expression changes and progression-associated signals in COAD, but these bulk-level results could not resolve the cellular source of *L1CAM*. Single-cell analysis placed L1CAM-associated signals within stromal and epithelial cellular contexts, rather than within T/NK cells themselves. These findings should be interpreted as exploratory and hypothesis-generating, providing a framework for subsequent spatial and functional validation.

The cellular localization of L1CAM is important for interpreting its tumor-related meaning. L1CAM has often been discussed in relation to tumor-cell invasion, migration and metastasis [21,26,32,33], but our data suggest that bulk L1CAM signals in colorectal cancer may also include stromal-derived components. L1CAM-positive fibroblasts showed neural-like, myelination-related, wound-response and matrix-remodeling transcriptional programs. These enrichment terms should not be interpreted as evidence for neuronal contamination or direct detection of neural cells; rather, they indicate a neural-like or wound-response-like fibroblast transcriptional state. By contrast, L1CAM-positive epithelial cells showed RNA-processing and ribosome-related programs, suggesting that L1CAM-positive fibroblasts and epithelial cells represent distinct cellular states.

At the sample level, L1CAM-Fib+ tumors were associated with reduced T/NK cytotoxicity transcriptional features in GSE178341. This association was more consistent for cytotoxicity signature scores than for T/NK-cell proportions, suggesting that L1CAM-positive fibroblast-containing samples may be linked to altered immune-cell transcriptional states rather than simply to immune-cell abundance. However, this result is based on a rare-cell population: only 57 L1CAM-positive fibroblasts were detected, and L1CAM-Fib+ grouping was defined using a detection-based presence/absence rule. Therefore, the L1CAM-Fib+ classification should be interpreted as a sample-level association marker, not as evidence for an abundance-dependent mechanism.

Candidate ligand–receptor and in silico attenuation analyses further prioritized several fibroblast-to-T/NK axes, including Galectin, HLA-E, TGFB and ECM-related interactions. These analyses are consistent with a candidate stromal-immune communication model, but they do not demonstrate direct functional suppression of T/NK cells. The attenuation analysis was a signature-based computational sensitivity analysis, not an experimental knockdown, ligand blockade or causal perturbation. Thus, the nominated axes should be viewed as candidates for future testing rather than validated mechanisms.

The supplementary external analyses indicate that the association observed in GSE178341 is dataset-specific. In GSE231559, marker-inferred L1CAM-positive fibroblast proportion showed a positive, rather than negative, association with T/NK cytotoxicity score. The additional exploratory dataset GSE188711 similarly did not reproduce the negative direction observed in the main cohort, showing a weaker and non-significant positive trend. These discrepancies may reflect differences in cell-type annotation strategy, sample composition, tumor site, sequencing depth, stromal and immune-cell recovery, or other dataset-specific factors. Because the supplementary datasets required marker-score-based inference rather than harmonized cell-type annotations and did not reproduce the negative direction observed in GSE178341, they are better interpreted as exploratory assessments of L1CAM-positive fibroblast-like state detectability rather than independent validation of the immune association. These discrepancies highlight the need for independently annotated single-cell cohorts and spatial validation.

Several limitations should be considered. First, this study is based on public transcriptomic and proteomic datasets and does not include new tissue-level validation by immunohistochemistry, multiplex immunofluorescence, RNAscope or spatial transcriptomics. Second, functional experiments such as fibroblast-immune-cell coculture, ligand perturbation or *L1CAM* manipulation were not performed. Third, detection-based single-cell definitions are sensitive to sequencing depth, dropout and cell recovery, particularly for rare-cell populations. These limitations restrict the interpretation of the present findings to computational associations and candidate biological hypotheses.

Overall, our analyses suggest that L1CAM-positive fibroblast-associated stromal states may provide a cellular context for interpreting a dataset-specific association between *L1CAM* expression and sample-level T/NK cytotoxicity features in colorectal cancer. Rather than supporting a single tumor-cell-intrinsic model, the data indicate that L1CAM-associated signals should be interpreted together with cellular source, stromal state and microenvironmental composition. Future studies combining spatial localization, orthogonal protein/RNA validation and functional assays will be required to determine whether L1CAM-positive fibroblast-like states have reproducible biological relevance in the colorectal cancer immune microenvironment.

## Data Availability

This study used publicly available de-identified datasets. TCGA expression and clinical data were obtained from GDC and UCSC Xena. GTEx normal tissue expression data were obtained from the UCSC Xena Toil recomputed TCGA-GTEx dataset. Protein data were obtained from the CPTAC module of UALCAN. The main colorectal cancer single-cell RNA-seq dataset was obtained from GEO under accession GSE178341, and the supplementary external exploratory datasets were obtained from GEO under accessions GSE231559 and GSE188711.

## Supplementary Materials

The following supporting information can be downloaded at https://www.mdpi.com/article/10.3390/biomedicines14091900/s1, Figure S1: Exploratory analysis of L1CAM-positive fibroblast-associated states in external single-cell datasets. (A) Marker-score-inferred cell composition across all GSE231559 samples. (B) Number of L1CAM-positive fibroblasts in each GSE231559 sample. (C) Correlation between the proportion of L1CAM-positive fibroblasts and T/NK cytotoxicity score across GSE231559 samples. (D) Marker-score-inferred cell composition across GSE188711 samples retained for exploratory analysis. (E) Number of L1CAM-positive fibroblasts in each retained GSE188711 sample. (F) Correlation between the proportion of L1CAM-positive fibroblasts and T/NK cytotoxicity score across retained GSE188711 samples. Cell types were inferred using broad marker scores. Correlations were assessed using Spearman tests; Figure S2: Sensitivity analyses for in silico attenuation and L1CAM-Fib+ grouping. (A) Candidate ligand–receptor axis scores across 0%, 25%, 50% and 75% attenuation levels. (B) Integrated candidate ligand–receptor score across attenuation levels. (C) Model-estimated T/NK cytotoxicity score across attenuation levels. (D) Sample counts under alternative L1CAM-Fib+ grouping definitions. (E) T/NK cytotoxicity scores under alternative L1CAM-Fib+ grouping definitions. (F) Correlation between continuous L1CAM-positive fibroblast proportion and T/NK cytotoxicity score. Paired attenuation comparisons used two-sided Wilcoxon signed-rank tests. Group comparisons used two-sided Wilcoxon rank-sum tests, and continuous association was assessed using Spearman correlation.
