# Supplementary material for: An L1CAM-Positive Fibroblast-Associated Stromal State Is Associated with Reduced T/NK Cytotoxicity Features in Colorectal Cancer
Source: Biomedicines. 2026 Aug 26;14(9):1900. doi: 10.3390/biomedicines14091900 (PMC13604495; doi:10.3390/biomedicines14091900)
Supplement: Supplementary file 1 [file biomedicines-14-01900-s001.zip › biomedicines-4479052-supplementary.pdf]

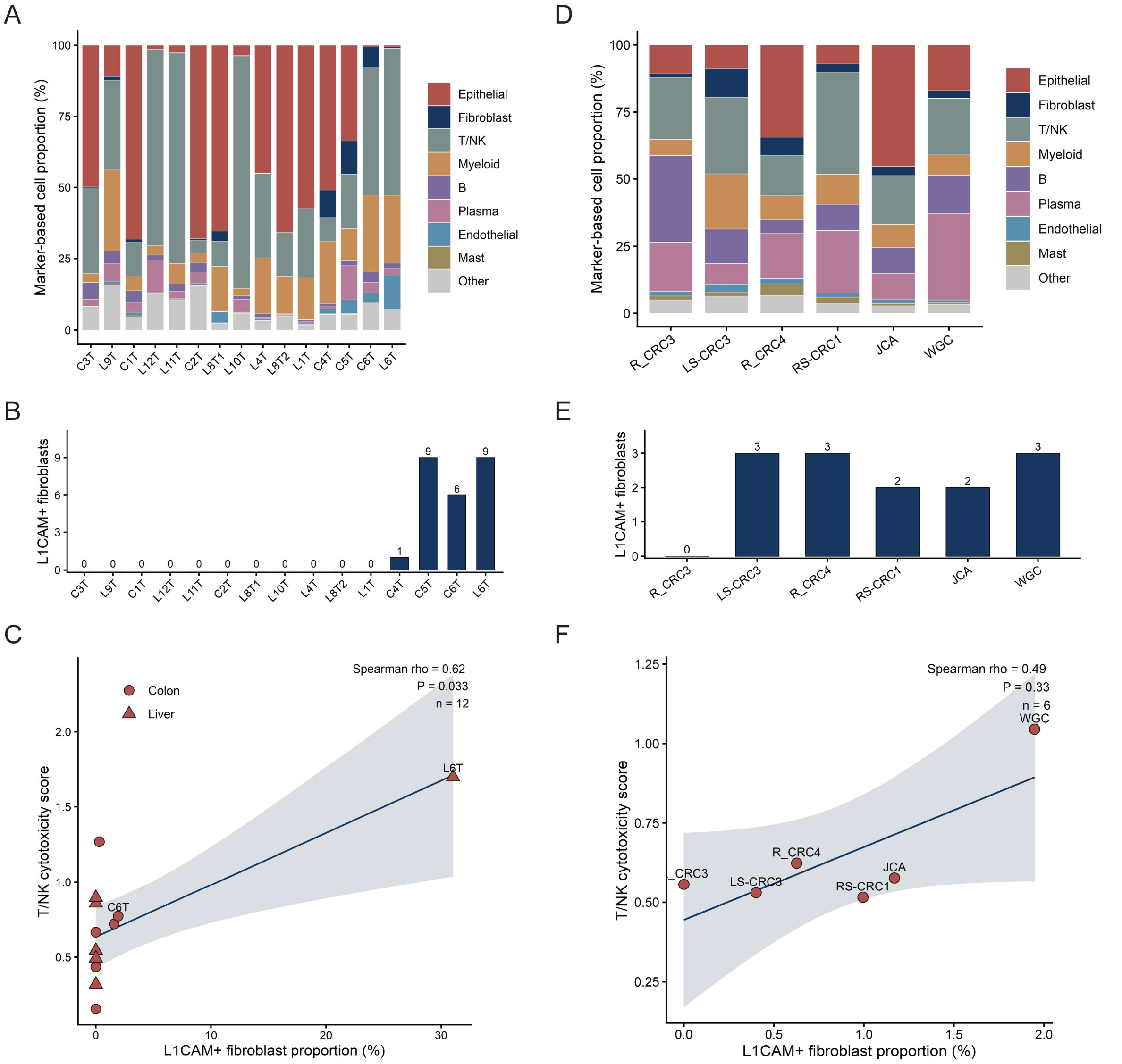

Figure S1. Exploratory analysis of L1CAM-positive fibroblast-associated states in external single-cell datasets. (A) Marker-score-inferred cell composition across all GSE231559 samples. (B) Number of L1CAM-positive fibroblasts in each GSE231559 sample. (C) Correlation between the proportion of L1CAM-positive fibroblasts and T/NK cytotoxicity score across GSE231559 samples. (D) Marker-score-inferred cell composition across GSE188711 samples retained for exploratory analysis. (E) Number of L1CAM-positive fibroblasts in each retained GSE188711 sample. (F) Correlation between the proportion of L1CAM-positive fibroblasts and T/NK cytotoxicity score across retained GSE188711 samples. Cell types were inferred using broad marker scores. Correlations were assessed using Spearman tests.

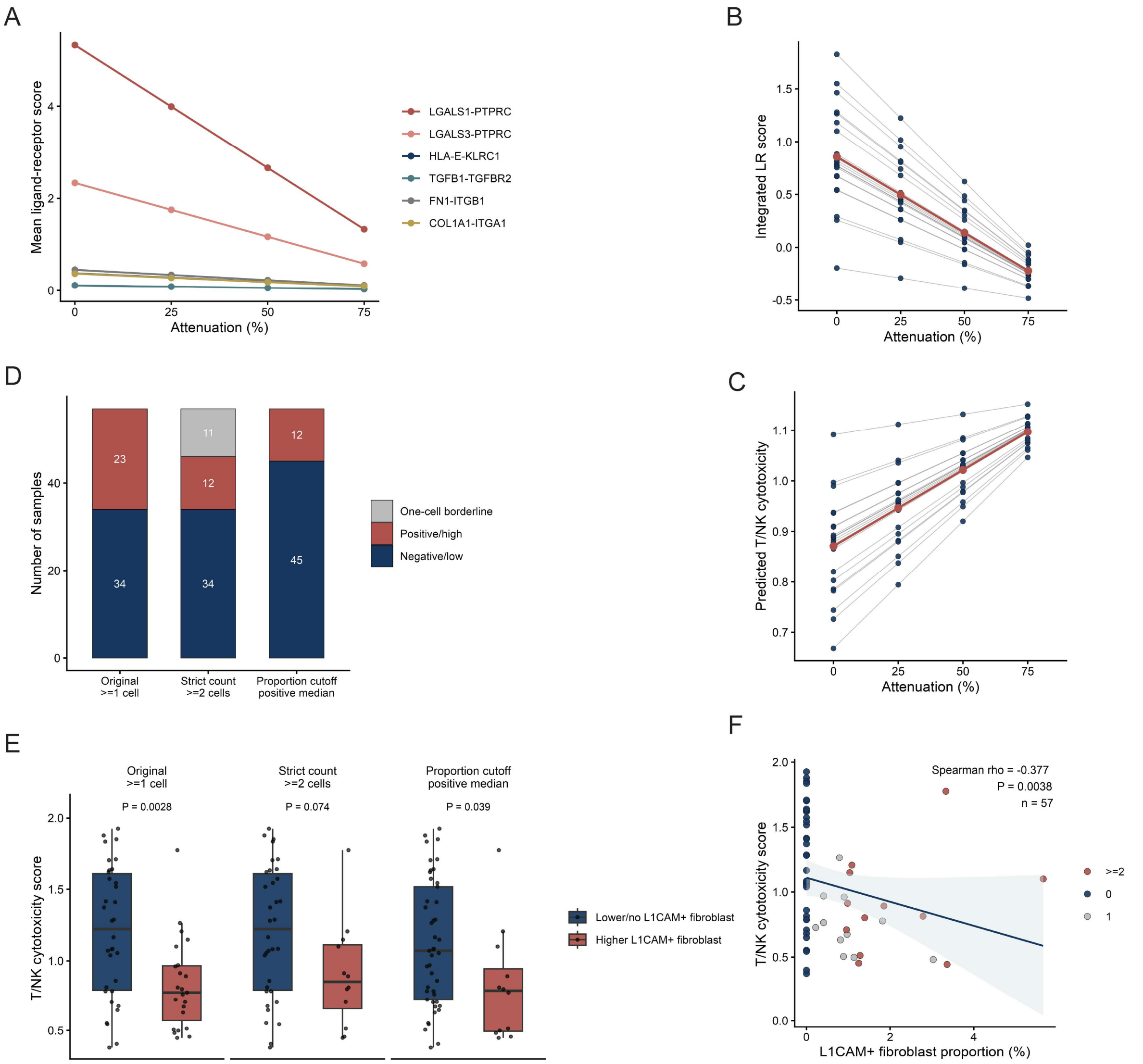

Figure S2. Sensitivity analyses for in silico attenuation and L1CAM-Fib+ grouping. (A) Candidate ligand–receptor axis scores across 0%, 25%, 50% and 75% attenuation levels. (B) Integrated candidate ligand–receptor score across attenuation levels. (C) Model-estimated T/NK cytotoxicity score across attenuation levels. (D) Sample counts under alternative L1CAM-Fib+ grouping definitions. (E) T/NK cytotoxicity scores under alternative L1CAM-Fib+ grouping definitions. (F) Correlation between continuous L1CAM-positive fibroblast proportion and T/NK cytotoxicity score. Paired attenuation comparisons used two-sided Wilcoxon signed-rank tests. Group comparisons used two-sided Wilcoxon rank-sum tests, and continuous association was assessed using Spearman correlation.
